# Artificial Intelligence and Multiple Sclerosis

**DOI:** 10.1007/s11910-024-01354-x

**Published:** 2024-06-28

**Authors:** Moein Amin, Eloy Martínez-Heras, Daniel Ontaneda, Ferran Prados Carrasco

**Affiliations:** 1 Mellen Center for Multiple Sclerosis Treatment and Research, Cleveland Clinic, Cleveland, OH USA; 2 Neuroimmunology and Multiple Sclerosis Unit, Laboratory of Advanced Imaging in Neuroimmunological Diseases, Hospital Clinic Barcelona, Institut d’Investigacions Biomèdiques August Pi i Sunyer (IDIBAPS), Universitat de Barcelona, Barcelona, Spain; 3 e-Health Center, Universitat Oberta de Catalunya, Barcelona, Spain; 4 Queen Square MS Centre, Department of Neuroinflammation, UCL Institute of Neurology, Faculty of Brain Sciences, University College London, London, UK; 5 Center for Medical Image Computing, University College London, London, UK; 6 National Institute for Health Research Biomedical Research Centre at UCL and UCLH, London, UK

**Keywords:** Multiple sclerosis, Artificial intelligence, Machine learning, Neurology, Data science

## Abstract

In this paper, we analyse the different advances in artificial intelligence (AI) approaches in multiple sclerosis (MS). AI applications in MS range across investigation of disease pathogenesis, diagnosis, treatment, and prognosis. A subset of AI, Machine learning (ML) models analyse various data sources, including magnetic resonance imaging (MRI), genetic, and clinical data, to distinguish MS from other conditions, predict disease progression, and personalize treatment strategies. Additionally, AI models have been extensively applied to lesion segmentation, identification of biomarkers, and prediction of outcomes, disease monitoring, and management. Despite the big promises of AI solutions, model interpretability and transparency remain critical for gaining clinician and patient trust in these methods. The future of AI in MS holds potential for open data initiatives that could feed ML models and increasing generalizability, the implementation of federated learning solutions for training the models addressing data sharing issues, and generative AI approaches to address challenges in model interpretability, and transparency. In conclusion, AI presents an opportunity to advance our understanding and management of MS. AI promises to aid clinicians in MS diagnosis and prognosis improving patient outcomes and quality of life, however ensuring the interpretability and transparency of AI-generated results is going to be key for facilitating the integration of AI into clinical practice.

## Introduction

Multiple sclerosis (MS) is a heterogenous chronic autoimmune condition of the central nervous system with associated neurodegeneration and when untreated results in significant disability. Clinical onset of MS is typically seen between the ages of 20 to 40 years and is thought to represent one of the most common causes of non-traumatic permanent disability in young adults. [1, 2] The incidence and prevalence of MS varies geographically and it affects nearly 1 million in the United States and approximately 2.8 million people worldwide. [3] The pathophysiology of MS is complex and likely multifactorial but it is thought to arise in genetically susceptible individuals with various exogenous factors including Epstein Barr Virus infection. [4, 5]

The current diagnostic criteria for MS are codified in the 2017 McDonald criteria. [6] The most recent revisions of the diagnostic criteria have placed an emphasis on increasing the sensitivity to allow an earlier diagnosis. [7] With the introduction, advancement of technology, and increased availability of magnetic resonance imaging (MRI), neuroimaging has become an important tool in diagnosis and monitoring of MS and is routinely used in clinical practice. [8] Although historically the diagnosis of MS relied on clinical features supported by MRI, blood tests, and cerebral spinal fluid (CSF) tests, the reliance on MRI has grown, so that now making a diagnosis without MRI features suggesting MS is the exception.

MS is a highly heterogeneous disease, and the clinical manifestations of MS can vary significantly, involving a variety of neurological symptoms including sensory, motor, visual, cognitive, and psychological changes. The multiple manifestations of MS makes following the disease over time challenging and requires incorporation of various clinical, laboratory, and radiological data.

In addition, MS clinicians may have access to a variety of other validated or investigational tools to assess for the disease and monitor for activity and complications including longitudinal neuro-performance measures, serum and CSF biomarkers, imaging biomarkers, electrodiagnostic data, neuropsychological assessments, patient reported outcomes, and optical coherence tomography (OCT). [9–14]

When considering longitudinal care, this can frequently lead to a significant number of data points available for a single person. For clinicians, the interpretation and significance of each individual data point may be challenging, and certain patterns may not be perceived readily without using sophisticated models.

The potential application of artificial intelligence (AI) in MS is particularly attractive for ongoing unmet needs. Topics where AI may provide solutions include issues related to misdiagnosis and late diagnosis through incorporation of MRI and blood biomarkers. Once the diagnosis is established AI may help to identify highly reliable prognostic markers, help identify treatment for progressive MS and aid in development of therapies that foster repair. Use of machine learning (ML) models in this setting can be optimal as they allow autonomous learning and pattern recognition from large datasets. Although ML models can be applied to any of the data types in MS care, their use in neuroimaging and MRI has been more extensively evaluated given the available data and the relatively standard format of imaging techniques across patients.

In this review, we present an overview of AI/ML in the context of potential applications in MS. Given the rapidly increasing number of publications in this field, rather than presenting a systematic/literature review, we present several select publications to introduce concepts of AI/ML to clinicians. These examples were chosen based on the relevance of the methods to the specific ML topic that is being discussed and highlight the current unmet needs in MS.

## Overview of AI

**Fig. 1:**
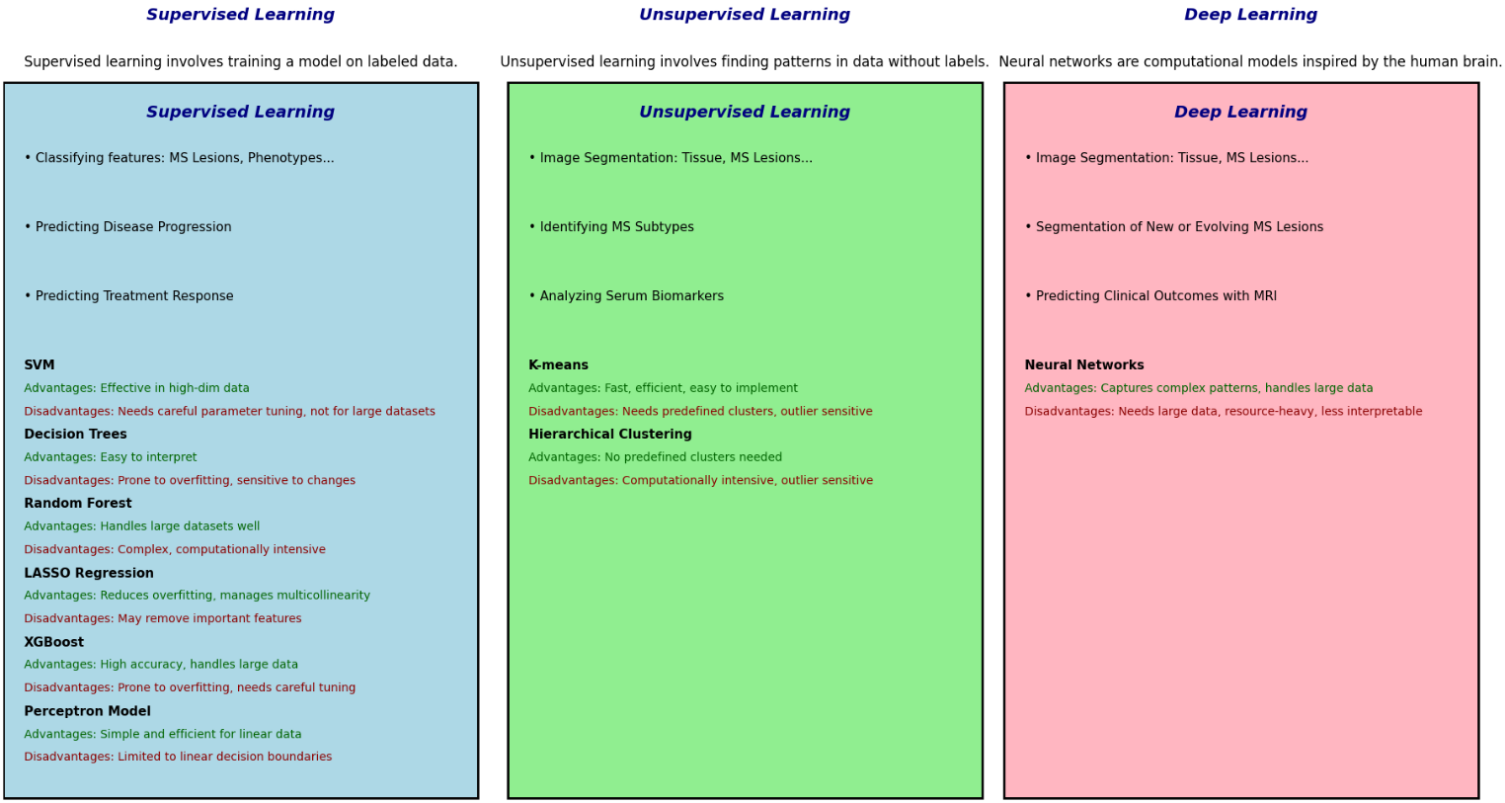
Summary of common machine learning categories and methods used in the multiple sclerosis field. Several examples of multiple sclerosis related research in each category are listed although this list is neither exhaustive nor exclusive. Advantages and disadvantages for each model are also summarized

Data science has rapidly advanced over the past century with increased availability of technology allowing larger data storage, faster processing, and lower costs. [15] This has led to significant progress in development of tools and methods in the fields of AI and ML. Although there is no commonly accepted consensus on the definition of AI, it is generally used to describe techniques that enable machines to learn from experience, recognize patterns, and perform tasks that are typically associated with human intelligence such as classification, inference, and prediction. [16] While AI represents a broad concept aimed to simulate human cognitive functions, ML is a subset of AI that specifically focuses on developing algorithms and statistical models that enable performance of specific tasks by learning from and making predictions or decisions based on data, essentially achieving autonomous learning. [17] In ML, algorithms inherently allow for self-improvement from experience and facilitate autonomous learning through generative training models to make useful predictions or generate content. [18] ML models typically follow one of the following AI domains: supervised learning, unsupervised learning, reinforcement learning, and generative AI. [18] Various methods fall under each of these domains and can be utilized depending on the task as each have advantages and disadvantages (Table 1; Fig. 1). [18, 19]

**Table 1:** Various ML methods

	Description	Typical applications
Supervised learning	The algorithm is designed to learn from a set of inputs and known paired outputs during a process called training or fitting the model. Once trained, this model can then be applied to unseen inputs to generate predicted outputs.	Classification (predicting categorical variables) or regression (predicting continuous variables) problems.
Unsupervised learning	The algorithm is designed to identify or recognize patterns in the input data without being provided with explicit output targets.	Grouping of complex variables without gold standard, using techniques like clustering.
Reinforcement learning	The algorithm is designed to produce an action when given a configuration of dynamic variables. The change on the variables after the action is then used to fine-tune the model, allowing the algorithm to learn strategies that maximize long-term optimization.	Processes that require long-term or delayed reward rather than immediate reward such as learning to apply certain rules in object detection.
Generative AI	The algorithm is designed to learn from patterns and structures of input data and generate new data with similar characteristics.	Generation of novel data in various mediums (such as text, image, video, etc.).

In ML, train/test splitting is common practice (commonly used in supervised learning). With this method, the models are trained and tested in different datasets, to ensure they generalize well to new, unseen data. A common approach is to divide the initial dataset into independent training, validation, and testing datasets randomly. The training dataset, which is typically larger than the others, is generally used to find the optimal model structure. The hyperparameters (model specific parameters that configure the learning process) are tuned based on model’s performance in the validation dataset. This validation dataset is essential for ensuring the model is adjusted correctly before final testing. The model’s final performance is then evaluated in the testing dataset, which is ideally an external and independent cohort, to ensure generalizability or to tune hyperparameters. Many ML models are prone to overfitting, which is an undesirable behaviour due to the model closely matching the training dataset, negatively impacting its performance on new external test dataset. This can be thought of as the model learning the specific features and noise in the training data rather than identifying the underlying patterns or rules governing the data. To detect this, evaluation of the model in an external testing or validation dataset is crucial, and to mitigate it, a heterogeneous training dataset is necessary.

Over the past several decades, there has been an increasing focus in integrating various ML methods in healthcare systems. With the integration of electronic health records, healthcare providers have been able to collect and store vast amounts of clinical, laboratory, imaging, genetic, and electrodiagnostic data, which may be challenging to interpret using traditional statistical models. AI and ML fields present a unique opportunity to take advantage of these large datasets to create clinically useful models. There have been several successful applications in various medical fields including radiology, pathology, cardiology, gastroenterology, ophthalmology, dermatology, oncology, and neurology. [15, 20, 21] Specifically, within the field of neurology there have been significant focus on using ML to analyse and interpret neuroimaging, electrodiagnostic, and genetics data. [19, 22, 23] Some examples include using ML to evaluate neuroimaging data during stroke evaluation, [24] neurodiagnostic and genetic data in neuro-oncology, [25, 26] electrodiagnostic and clinical data in epilepsy, [27] video evaluation of movement disorders, [28] among others. To overcome inherent data limitations such as data sparsity, small sample sizes, and high dimensionality, researchers have increasingly participated in challenges. These competitions facilitate the comparison and development of innovative ML models within controlled environments by providing access to larger, more diverse datasets and fostering collaborative solutions to complex problems.

## AI Applications for Investigation of MS Pathogenesis

MS pathogenesis is described by the inflammatory response of white and grey matter tissues in the CNS. AI technologies can help to enhance our understanding of these processes by analysing large datasets to identify patterns that might not be visible. There are different biomarkers used to diagnose MS like serum levels, CSF markers, neuroimaging and other approaches. AI can assist in the interpretation of these biomarkers, potentially leading to more accurate diagnoses and personalized treatments. Whilst supervised and unsupervised solutions are currently the preferred approaches, over the next few years we are going to see an increase in the number and quality of the solutions based on reinforced learning or that benefit from generative AI approaches. Also, it is important to highlight that the sophistication and precision of the approaches will increase with the volume of data available. In the following subsections, we discuss various AI applications in biomarkers for MS diagnosis.

### Serum Levels

Understanding the underlying systemic changes in MS is a vital process in understanding the pathophysiology of MS. In a recent study conducted in Brazil, [29] authors used several supervised ML models to study the performance of serum levels of various antioxidants in distinguishing MS from healthy controls (HC). Authors initially used a binary logistic regression model using all predictors which showed 4 antioxidant levels (zinc, adiponectin, total radical-trapping antioxidant parameter, and sulfhydryl groups) were amongst most important features distinguishing MS vs. HC. In a logistic regression, the model is trained to predict the probability of a sample belonging to a certain category based on several input variables using the form below. The probability $p$ that sample X belongs to category 1 is calculated using the coefficients β:

$$p\left(Y=1| X\right)= \frac{{e}^{{\beta }_{0}+{\beta }_{1}X}}{1+{e}^{{\beta }_{0}+{\beta }_{1}X}}$$

Here, β0​, β1​,…,βn​ are the coefficients that the model learns, and X1​,X2​,…,Xn​ are the input variables.

**Fig. 2:**
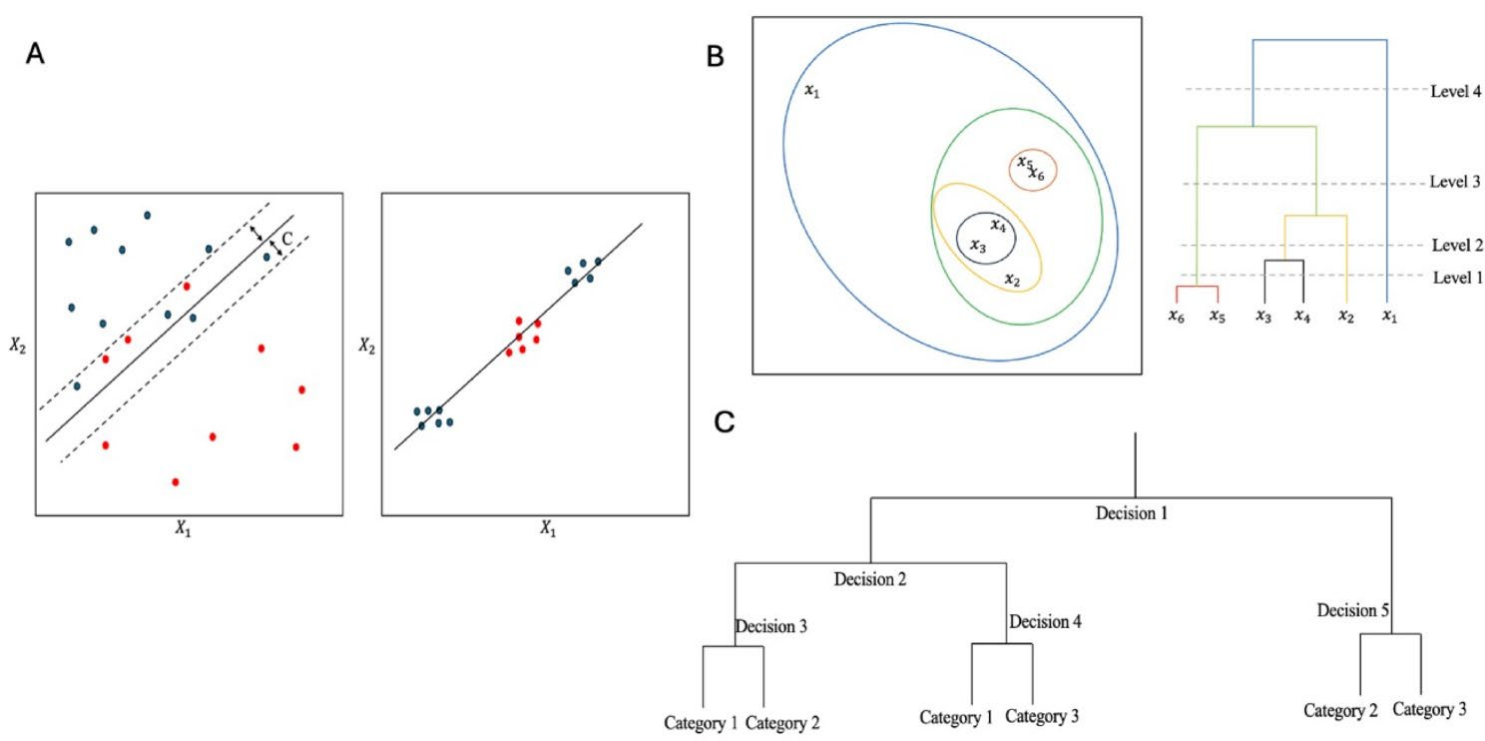
Examples of ML methods. **Panel (A)** Examples of how SVNs may help classify data in 2D. In the left panel, noise in data creates inaccuracies when using a linear decision boundary such as in a logistic regression model. The parameter C in the SVM model allows for flexibility in the decision boundary. In the right panel a linear decision boundary is unable to separate data. A polynomial or radial kernel may be able to separate the data better in the SVM model. **Panel (B)** Hierarchical clustering of 6 samples shown in the left panel. The right panel represents the dendrogram which can be cut at different levels (level 1, level 2, level 3, or level 4) to categorize various numbers of clusters. **Panel (C)** An example of a decision tree where a category is determined based on a combination of decision rules

Given that a limitation with logistic regression models is their linear decision boundaries, the authors also utilized more advanced ML models that can define non-linear relationships. Specifically, they employed support vector machine (SVM) and neural networks (NN), which are capable of holding more complex patterns in data. These models will be discussed in further detail below and using these the authors demonstrated a correlation between reduced levels of systemic antioxidants and probability of MS (vs. HC) suggesting a possible role for these antioxidants in the pathogenesis of the disease although these results will require further examination and validation. Similar to logistic regression, SVM also aims to find a decision boundary based on a combination of variables to separate cases and controls. However, one distinct difference is that SVM allows for noise in the data by introducing a regularization parameter C. This parameter can be adjusted to produce different models which each allow different levels of allowances for observations to be on the incorrect side of the decision boundary. In addition, SVMs can use different types of mathematical functions, known as kernels, which help handle more complex scenarios when separating the data (Fig. 2, A). This includes data that may not be linearly separable, such as polynomial, sigmoid, or radial decision boundaries. This finding underscores the capability of advanced ML techniques to uncover complex relationships within medical data that may not be readily apparent with simpler models. Although SVMs offer additional flexibility for selecting a decision boundary, they achieve this by increasing the dimensionality (and complexity) of input features, which reduces interpretability. Although some methods exist to visualise the impact of change in each parameter in the overall predictions of an SVM model, the computed coefficients may not be as readily interpretable as in a logistic regression model, where coefficients may be interpreted in the context of log odds of probability.

**Fig. 3:**
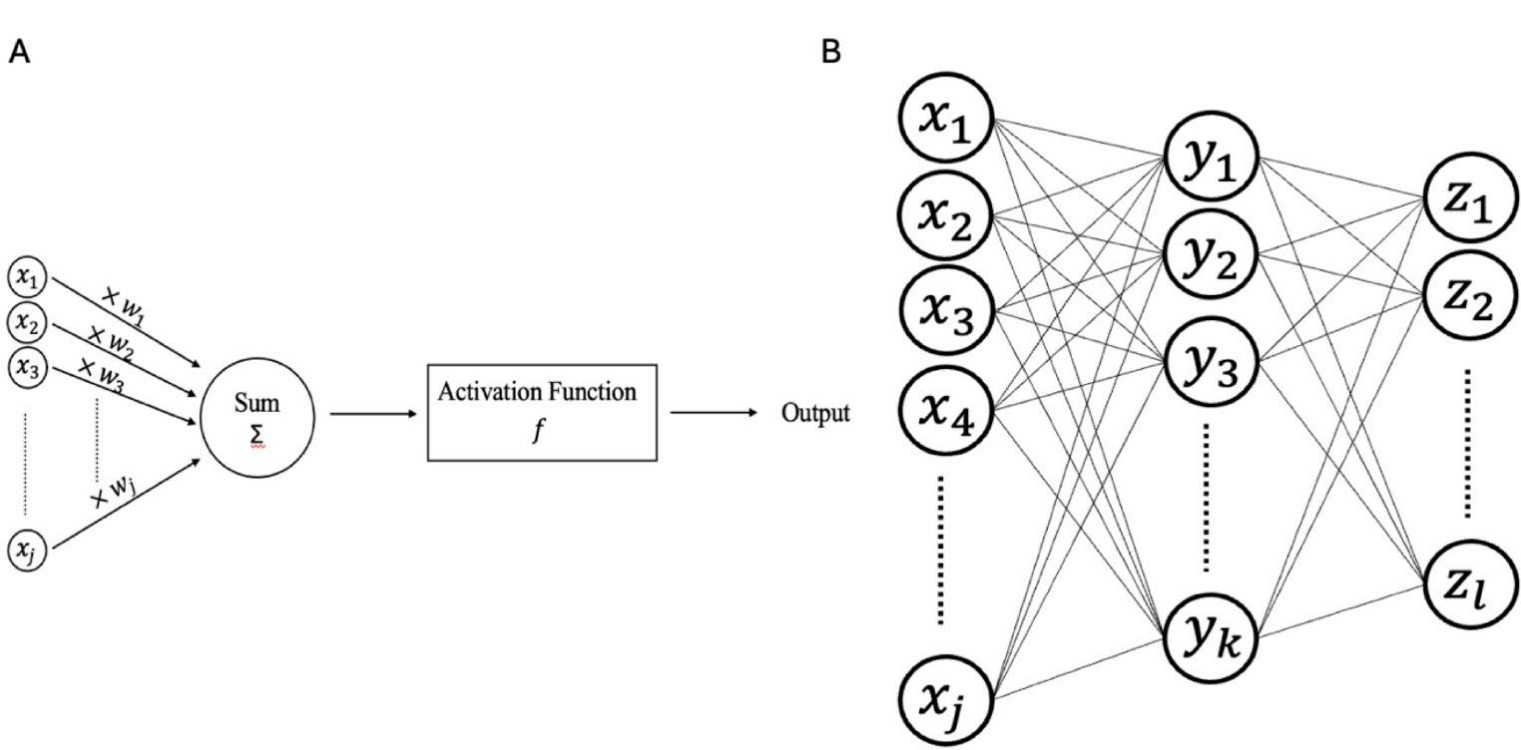
Neural networks. **Panel (A)** Typical structure of a perceptron consisting of one neuron. **Panel (B)** NNs are built from several different perceptron connected to each other through layers

The alternative method involving NNs can be best understood as an advanced form of a simple perceptron model. A perceptron can be thought to mimic a single neuron, where the model receives multiple inputs (${x}_{1}, {x}_{2},{x}_{3},\dots , {x}_{j}$) with each having a different weight (${w}_{1}, {w}_{2},{w}_{3},\dots , {w}_{j}$). The neuron then applies an activation function f on the input to produce an output (Fig. 3, A). Compared to SVMs, NNs can fit models with more complex interactions between the input parameters. This is because NNs have the inherent flexibility to implement multiple layers between the input and the output layers, where each layer iteratively combines and manipulates elements from the preceding layers. Additionally, unlike SVMs, NNs do not require assumptions about the underlying relationship between the input parameters. This feature is particularly useful when modelling high dimensional data or data where the relationship between input and output is unknown or not well defined. However, a potential downside of using more complex models such as NNs over SVMs is the possibility of overfitting or fitting to noise instead of the underlying true pattern, though with proper regularization and validation techniques, the models created in NNs can be generalizable.

The process of training for a perceptron includes randomly initializing all the weights (w) and then computing the produced output using a training example. The difference between the true output and the produced output by the perceptron is used as a penalty to provide feedback and update the weights. This process is repeated several times until the weights are optimized to minimize the penalty. A neural network represents a more complex from of a perceptron, comprising multiple neurons connected to each other in different layers (Fig. 3, B). Use of more advanced ML techniques in the work discussed above allowed exploration of more sophisticated relationships in prediction of outcome and improved accuracy but this may come at a cost of reduced generalizability and interpretability which may limit our ability of inferring causal relationships or identifying a biomarker for further evaluation in future studies. Over the last few years, other AI applications that used serum levels in different ways have been proposed for MS prognosis with some further examples including to develop predictive model of retinal layer changes using serum neurofilament light chain (sNfL) [30] and predictive model of cognitive changes using sNFL. [31]

### CSF Markers

In a recent publication, 92 CSF biomarkers were studied in MS and patients with other neurological diseases (OND). [32] Authors initially used an unsupervised ML technique called hierarchical clustering to evaluate for interactions and correlations between various proteins and divide proteins into clusters. Hierarchical clustering is a bottom-up clustering approach where each observation starts as its own cluster and iteratively the two closest clusters are merged to form a new cluster until all observations are in one cluster. The results can be presented in a dendrogram format which can be used to establish various numbers of clusters (Fig. 2, B).

In the study of examining CSF biomarkers, logistic regression was used to evaluate the predictive ability of these biomarkers to distinguish MS from OND. [32] In this model, the coefficient for each variable (β) indicates the weight or importance of that variable in predicting the outcome of interest. However, comparison of β for different variables should be done cautiously as β coefficients is also influenced by the units and distribution of each variable. For meaningful comparisons, all variables should be scaled. A significant problem with a logistic regression model is the feature selection; training model with a large number of variables can lead to overfitting. To address this, a common strategy is to select a subset of predictors. One known approach for feature selection that was employed in this paper is using penalization or regularization to prevent overfitting in the logistic regression model. [32] Specifically, LASSO regression or L1 regularization was employed, which minimize the total sum of β coefficients, potentially reducing some coefficient to zero and thus eliminating them from the model to reduce the number of variables used in the model (although interpretations of removed variables require caution). Using this model, the authors successfully identified several biomarkers effective for differentiating MS from OND. [32] The use of hierarchical clustering was useful in the work presented above to allow creation of clusters of proteins correlating with each other. However, as this is a form of unsupervised learning, it requires human intervention on selection of criteria for stopping the clustering and determining the number of clusters. Often, different criteria need to be examined to find the optimal solution.

Other examples of ML predictive models distinguishing MS from mimics using CSF cytokines have been developed over the recent years. [32, 33]

### Neuroimaging Features

In one study, authors used diffusion tensor metrics to evaluate their association with future disability. [34] In this study, LASSO regression was used which can potentially help in dealing with multicollinearity between several MRI parameters. As a result, they were able to identify important regions of interest where grey matter measurements and functional connectivity significantly impacted disability. [34] Although LASSO regression is a useful method to create a predictive algorithm to avoid overfitting when using datasets with large number of predictors with possible multicollinearity, it is important to note that interpretation of feature coefficients from LASSO regression has to be done with caution as this method may select or remove highly correlated variables randomly. Furthermore, the shrinkage effect of LASSO can lead to underestimating the importance of some predictors.

Other unsupervised clustering methods have also been explored to study MS heterogeneity. In a study aiming to classify MS subtypes based on pathological features, [35] authors analysed quantitative MRI features from several large MS datasets to identify three distinct clusters: cortex-led, lesion-led, and normal-appearing white matter-led. They demonstrated that these clusters were associated with different probability of clinical outcomes, including disease progression and response to treatment. Similar to limitations discussed in previous work utilizing unsupervised learning, this mode of learning requires human input when selecting the number of clusters or subtypes. Authors in this work utilized cross-validation to identify the optimal number of subtypes to be used in model development.

In recent years, AI solutions using neuroimaging markers have been extensively developed to elucidate the complexities of MS disease. There are efforts for generating tools to improve the resolution of the MRI scans, [36] applying deep learning to compute synthetic images or generate missing contrast, [37, 38] and employing generative AI solutions for image segmentation or synthetic data generation. [39, 40] There are also initiatives to obtain global values or biomarkers from the whole image like predicted brain age difference (BrainPAD), [41, 42] using graph neural networks or graph-based convolutional networks. [43, 44]

### Other Domains

The genetic basis of MS has long been a topic of interest. Although the methods used in these studies are beyond the scope of this manuscript, it is important to note that both unsupervised and supervised ML models plays an important role in analysing large genetic datasets. [45–47]

## AI Applications in MS Diagnosis

Despite recent advancements in MRI technology and revisions to the McDonald criteria, misdiagnosis rates in MS remain high, primarily attributed to lack of specific biomarkers and neuroradiological mimics of MS. [48–50] ML can be a valuable asset in this area, as it enables the development of highly accurate predictive models that can help distinguish MS from others pathologies based on high dimensional data, which may be challenging to analyse using more traditional statistical methods. For example, convolutional neural networks (CNN) have been used not only to distinguish MS from HCs, [51] but also to differentiate MS from highly prevalent radiological mimics. [52, 53] Given the limitations of neurological history and examination in accurately identifying all relevant changes in clinical status, ML models to evaluate alternative sources of clinical data have become valuable. [54] In addition to MRI data, other clinical data can be used in training ML models to distinguish MS. In a study evaluating speech data recorded from patients with MS, neural network approach was able to differentiate MS from HCs and this approach outperformed traditional digital speech signal processing approaches. [55] In this study, audio recordings from participants repeating syllables as accurately and quickly as possible were used as input to each of the models. With recent innovations in wearable sensors and technology, these devices have become increasingly available and affordable and thus their use could be leveraged to augment data collected during clinical visits. [56] ML methods have been used to analyse various parameters collected through wearable or mobile sensors to monitor gait and ambulation in MS and offer contrast to distinguish them from HCs. [57, 58]

**Fig. 4:**
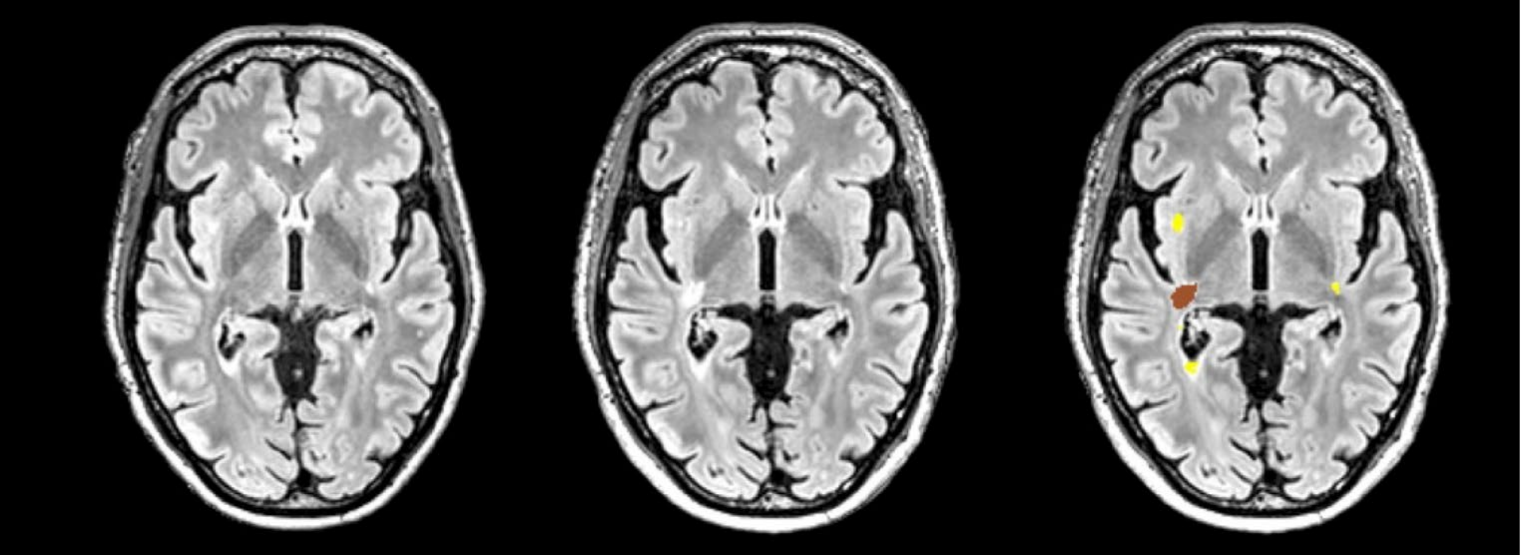
3D-FLAIR sequences demonstrating the detection of new MS lesions using nnU-Net architecture. Lesions identified at baseline are marked in yellow, while the brown lesion indicates an interval new lesion

One of the first and most well-established ML methods in MS neuroimaging analysis involves MRI lesion segmentation, employing a variety of automated techniques. Segmenting lesions in MRI scans has been pivotal for both diagnosing and monitoring the progression of the disease. Historically, emphasis was placed on cross-sectional images, but this shifted in 2017 with the revised McDonald criterion, highlighting the need to evaluate disease progression spatially and temporally. By 2020, the methodology landscape evolved significantly towards advanced deep learning techniques. [59] The introduction of the Open MS Data dataset [60] and the MSSEG-2 challenge dataset helped standardize benchmarks, consisting of conventional MRI data from multiple centres using diverse scanners to develop robust models. Most entries in the MSSEG-2 challenge employed CNNs, with U-net architectures proving popular. [61] The nnU-Net v2 architecture, with its hierarchical labelling, further refines the ability to segment complex structures, aiming to enhance segmentation precision and adaptability in recognizing new and basal lesions, thus addressing challenges of variability and inconsistency in MRI acquisitions (Fig. 4). Other architectures based on the 3D-CNN have also been applied to identification of biomarkers such as the central vein sign. [62]

## AI Applications in MS Prognosis

Individualising care in medicine, including neurology, is a topic with continuously increasing demand, and MS is no exception. Currently, most prognostic models are based on population level data, and given the significant heterogeneity of the disease, drawing inference to the individual level becomes a challenge. ML models provide an opportunity to incorporate large datasets and provide more accurate prognostic information applicable to individuals. One such tool is use of digital twins whereby multidimensional data representing various aspects of patient’s data are collected digitally including relevant biological, anatomical, and physiological parameters which can then be used to predict response and prognosis. [63] Several studies have also attempted to evaluate the ability of genetic data in predicting future risk of disability which has led to concept of CNS resilience in progression of MS. [47, 64, 65] In one study, [66] postural and ambulation data recorded were used to predict risk of future falls in MS. In this study a supervised ML method frequently used in categorisation problems referred to as random forest was employed to determine the subsequent risk of falls (low, moderate, high). A random forest model is based on a tree decision tree model which uses recursive binary splitting with decision rules to categorize observations (Fig. 2, C). A random forest model is a variation of a decision tree such that several different iterations of trees are trained by taking repeated samples from training dataset using bootstrapping and at each split a random selection of predictors can be considered. The final categorisation is made using majority voting across all the trees. This method effectively improves prediction accuracy and reduces the likelihood of overfitting by averaging multiple decisions from diverse trees. Using random forests to make predictive models can be useful, as this method can counteract the variability and noise encountered in simple decision trees models. However, this model can have limited interpretability and susceptibility to overfitting particularly when using a relatively small sample size as was done in this work. Therefore, the generalizability and validation in other datasets must be scrutinized.

A variation of random forest model is another tree-based model called XGBoost which was used in a different study predicting risk of disease activity in patients treated with cladribine based on data from clinical trials. [67] This method takes advantage of boosting, which like random forest, involves training several times. However, unlike random forest, where each tree is trained independently, boosting trains each tree sequentially. Each successive tree in boosting learns from the errors of the previous ones, thereby improving the accuracy of the model over iterations. Similar to other tree-based methods, XGBoost is prone to overfitting, particularly with small datasets and a large number of parameters. Therefore, results usually require external validation.

Additionally, other clinical parameters including electrodiagnostic data have also been used in predicting clinical symptoms and disability in MS using methods previously described. [68, 69]

In MS, over the last decade, AI neuroimaging solutions have been introduced in the form of cross-sectional and longitudinal quantitative volumetric reports, which have been commercialized by several AI-radiology-based companies. [70] These quantitative volumetric reports are very useful because they contextualize a single subject’s results in comparison to a normative database [71–74] for the most common MS biomarkers (i.e. lesion volume, lesion count, or brain atrophy among others). A recent systematic review by Mendelsohn et al. [70] found up to 38 relevant publications using MS quantitative volumetric reports developed by 10 different companies. This review highlighted some key steps for the widespread adoption of these reports: clear clinical validation and also end-user testing.

## Current Challenges and Limitations

Perhaps, the most important current challenge in MS is having access to large datasets for training robust AI models to capture the full spectrum of disease variability. This is exceedingly challenging considering the current ethical and compliance frameworks, which demand the creation of large, standardized datasets including MRI, blood, and other clinical information. These freely available datasets are crucial for validating MS outcomes, particularly those related to neurodegeneration and disease progression. Consequently, there is a need for a more comprehensive and efficient approach to study MS progression, including possibilities to distribute data to a broad base of sites capable of developing powerful AI solutions. In response to the growing movement towards open science, the scientific MS community must make an effort to create accessible datasets for accelerating research progress and increase the generalizability of newer AI methods. Open science promotes collaboration, transparency, and broader data accessibility, advancing scientific discoveries and innovation. Moreover, the FAIR principle (Findable, Accessible, Interoperable, and Reusable) underscores the importance of making data publicly available to maximize its usefulness across various research fields. Although several efforts have been made in establishing open MS datasets, the availability of large public repositories containing MR data remains limited. [60, 75]

Most of the current AI models are still black boxes despite several research efforts attempting to elucidate these aspects. Ensuring interpretability and transparency of AI-results are crucial for fostering trust among clinicians and patients. In the clinical setting, we need to incorporate clear procedures. In this regard, transparent AI algorithms will provide insights into how decisions are made, aiding clinicians in understanding and validating results. Meanwhile, interpretable AI-results will enable clinicians to identify potential biases or errors, enhancing the interaction human-machine and increasing the accuracy of diagnosis and prognosis in MS. By easing interpretability and transparency, AI applications will have a smoother path for their translation and use into the real clinical setting, ultimately enhancing patient care.

Many studies evaluating ML models in diagnosing MS perform well when the comparison cohort is done to healthy controls and in patients with established diagnoses. For ML models to become more practically integrated in clinical practice and address the unmet clinical needs, these models need to be trained and validated in patients in early stages of disease (and pre-clinical stage) at the time of diagnostic evaluation and diagnostic performance needs to be optimized compared to other radiological or clinical mimickers.

The ethical concerns surrounding the use of AI have been a source of debate and their use in neurology and MS carries similar concerns. ML models often perform well in the datasets in which they are trained. However, if certain populations are less represented in these datasets, subsequent models may not be valid to be used. Furthermore, the use of AI may imply less accountability given that human interaction is reduced, and decisions based on AI models can be difficult to scrutinize. Possibility of intellectual ownership of ML models is also a source of ethical concern, as it may lead to the monopolization of methods and datasets and reduce collaboration.

## Future Outlook

The outlook of ML applications in promises numerous opportunities to address unmet clinical needs. As discussed previously, one of the limitations in application of ML in MS is easy access to large, diverse, and open datasets. Access to such datasets would allow training of more generalizable models and more accurate validation of models. Although a limited number of such datasets exist, further collaboration within the MS community could facilitate this process. There have been several attempts for such collaborations related to MS and other medical fields, however, these have faced limitations that need to be further addressed in the future. [76, 77] One possible solution to some of the challenges in open medical data sharing is federated learning which involves collaboration of several centres without sharing the actual data used to train models but instead using a decentralized privacy-preserving technology to update models collaboratively and iteratively. [78] Beyond using conventional MRI features in training MS models, there has also been an interest in use of ML models applied to radiomics. These models have particularly been of interest when applied to differentiate MS from other radiological mimickers as well as understanding MS pathophysiology. [79–82] Generative AI models are still in their early and experimental stages in MS with numerous potential possibilities. One example of such technology is ChatGPT with one simple application including communicating with patients. [83] In this study, authors examined and compared the empathy scores between neurologist and ChatGPT responses, finding similar satisfaction scores but higher empathy scores with ChatGPT responses by the patients. This was possibly attributed to the more informal tone used in ChatGPT responses, and the potential work-load limitations faced by neurologists. Interestingly, those with higher education levels had lower likelihood of preferring ChatGPT responses compared to neurologist responses. This study highlights the potential utility of generative AI in bridging some gaps in communication and education with patients, offering relevant, simple, and real-time answers. However, integration of these tools into clinical practice requires further research and optimization due to current limitations, including inaccurate responses without clinician supervision and limited adaptability to personalised factors such as age and education.

## Conclusions

The translation of AI in the MS clinical setting is a great opportunity to revolutionize MS diagnosis and prognosis ultimately improving patient outcomes and care. Research studies showed that AI models have the potential to provide clinicians with valuable insights in decision support, efficiency, and effectiveness in patient care. As any other technology, more research on the field is going to increase robustness of the models and new initiatives for MS data sharing will be needed. With additional advances and increased availability of computational technology such as quantum computing, our ability to perform more sophisticated analyses on higher dimensional data holds promise for more individualised models. Moreover, the field needs to overcome challenges in interpretability and transparency in how the results are computed to gain the trust from clinicians, patients and have a smoother transition of these technologies into the real clinical setting. Over the next few years, we anticipate increased efforts for drawing and clarifying the clinical validation processes, regulatory compliance, ethical considerations, and the iterative improvement processes needed for any AI model in the clinical setting.

## Data Availability

No datasets were generated or analysed during the current study.
